# All-optical crosstalk-free manipulation and readout of Chronos-expressing neurons

**DOI:** 10.1088/1361-6463/aaf944

**Published:** 2019-01-09

**Authors:** Navjeevan S Soor, Peter Quicke, Carmel L Howe, Kuin T Pang, Mark A A Neil, Simon R Schultz, Amanda J Foust

**Affiliations:** 1 Centre for Neurotechnology, Imperial College London, London, United Kingdom; 2 Department of Bioengineering, Imperial College London, London, United Kingdom; 3 Department of Physics, Blackett Laboratory, Imperial College London, London, United Kingdom; 4 Institute of Molecular and Cell Biology, Agency for Science, Technology and Research (A*STAR), Singapore; da944em1 a.foust@imperial.ac.uk

**Keywords:** Chronos, calcium indicator, optogenetics, crosstalk, functional imaging, neuron, neuromodulation

## Abstract

All optical neurophysiology allows manipulation and readout of neural network activity with single-cell spatial resolution and millisecond temporal resolution. Neurons can be made to express proteins that actuate transmembrane currents upon light absorption, enabling optical control of membrane potential and action potential signalling. In addition, neurons can be genetically or synthetically labelled with fluorescent reporters of changes in intracellular calcium concentration or membrane potential. Thus, to optically manipulate and readout neural activity in parallel, two spectra are involved: the action spectrum of the actuator, and the absorption spectrum of the fluorescent reporter. Due to overlap in these spectra, previous all-optical neurophysiology paradigms have been hindered by spurious activation of neuronal activity caused by the readout light. Here, we pair the blue-green absorbing optogenetic actuator, Chronos, with a deep red-emitting fluorescent calcium reporter CaSiR-1. We show that cultured Chinese hamster ovary cells transfected with Chronos do not exhibit transmembrane currents when illuminated with wavelengths and intensities suitable for exciting one-photon CaSiR-1 fluorescence. We then demonstrate crosstalk-free, high signal-to-noise ratio CaSiR-1 red fluorescence imaging at 100 frames s^−1^ of Chronos-mediated calcium transients evoked in neurons with blue light pulses at rates up to 20 Hz. These results indicate that the spectral separation between red light excited fluorophores, excited efficiently at or above 640 nm, with blue-green absorbing opsins such as Chronos, is sufficient to avoid spurious opsin actuation by the imaging wavelengths and therefore enable crosstalk-free all-optical neuronal manipulation and readout.

## 1. Introduction

All-optical activation and readout of neuronal activity has the potential to revolutionise our understanding of neural network structure and function [1–3]. Neurons can be made to ectopically express light-sensitive ion channels or pumps (opsins) to control their activity [4–6]. Furthermore, they can be synthetically [7] or genetically labelled [8, 9] with activity reporters that convert biophysical markers of cellular activity such as changes in intracellular calcium concentration [10, 11] or membrane potential [8] into a change in fluorescent signal. These optogenetic technologies offer a complement to electrical modalities [12] such as patch clamp and extracellular electrophysiology by providing functional, spatial, and cell class information from networks of 10s–1000s of neurons with single-cell spatial resolution and millisecond temporal precision. Moreover, optical methods do not require direct mechanical contact with tissue. The ability to deploy and collect light to and from many neurons in parallel can enable access to the brain at network scales, and thus improve knowledge of how these networks participate in behaviour [13] and pathology [14]. Importantly, optical neurophysiology potentially enables causative, closed-loop studies of neural networks which were previously unattainable.

An issue yet to be solved in all-optical neurophysiology paradigms is that of optical crosstalk between the photostimulation and activity read-out optical pathways (recently reviewed in [15, 16]). If the opsin actuator is sensitive to wavelengths used to image neural activity, this can cause photocurrents which spuriously affect the studied network’s function. This is crucial as sub-threshold changes in membrane potential, those which do not reach action potential firing threshold, can increase local network weights [17] and enable changes in neuroplasticity [18]. Similarly, excitation of the fluorescent activity reporter by the photostimulation wavelengths can cause imaging artefacts on the readout channels. This latter form of crosstalk can be quantified and subtracted, and moreover does not affect network function.

The broad action spectra of optogenetic actuators [14] render overlap with fluorescent reporter absorption difficult to avoid. Efforts to reduce crosstalk in one-photon excitation schemes with large spectral overlap between actuator and reporter (e.g. the actuator channelrhodopsin-2 [ChR2]  +  GCaMP calcium reporters) have minimised readout light intensities [19, 20] to the detriment of signal-to-noise ratio (*S*/*N*). Szabo *et al* [20] also patterned the readout light over neuronal somata to reduce spurious actuation in dendrites and axons. One-photon, low spectral-overlap readout of ChR2-expressing neurons has been achieved with voltage reporters [21–24] and red-shifted genetically-encoded calcium reportors RCaMP [11], R-GECO [25, 26], CAR-GECO1 [27], RCaMP2 [28], jRGECO1a and jRCaMP1a/b [29]. Hochbaum *et al* combined the red genetically encoded voltage indicator QuasAr with the blue-shifted excitatory actuator CheRiff [30] with only subthreshold activation of CheRiff by QuasAr excitation light. While suitable for single-neuron, low spatial resolution population imaging, or cellular resolution imaging of neuronal networks in culture [31], the low specificity and fractional change in fluorescence over background (*dF*/*F*) of voltage reporters severely limits their application to cellular-resolution imaging of neuronal networks in mammalian brain slices and *in vivo* preparations. Red-shifted calcium reporters have the potential to address this gap with decreased temporal resolution but undergo photoswitching and fast photobleaching during blue light illumination, further discussed in [32]. Alternatively, it is possible to combine red-shifted actuators [5, 33] with green calcium reporters, though these proteins exhibit 20%–30% actuation efficiency under blue light excitation [34], and thus these pairings are more susceptible to spectral crosstalk than pairings where the actuator is more blue than the reporter.

All-optical systems based on two-photon excitation of actuators and reporters feature optical sectioning and improved depth penetration and robustness to scattering compared to one-photon excitation [35]. Unfortunately, crosstalk is more difficult to avoid in such systems due to broad two-photon spectra. Studies have combined one-photon excitation of ChR2 actuators with two-photon excitation of spectrally overlapping calcium reporters [36, 37], taking advantage of ChR2’s low two-photon excitation efficiency when scanned [38].

Previous studies have attempted to minimise crosstalk in various ways. Baker *et al* [39] targeted ChR2 to neuronal somata, which can reduce spurious actuation through axon and dendrite readout illumination. dal Maschio *et al* [13] excited two-photon GCaMP6s fluorescence at sub-optimal wavelengths to reduce spurious ChR2 actuation. Although they reported no significant increase in the firing rates of ChR2-expressing neurons when imaging GCaMP6s fluorescence, undetected subthreshold depolarisations were likely as ChR2 exhibits non-negligible two-photon absorption at the 1020 nm imaging wavelength [40]. Furthermore, this strategy reduces the *S*/*N* of the activity readout due to decreased reporter excitation rates at sub-optimal wavelengths. Another strategy to minimise stimulation crosstalk is fast scanning of the imaging light path over the region of interest (ROI) containing the opsin expressing cells. This was first demonstrated by Packer *et al* [41] with the red-shifted actuator C1V1 combined with the reporter GCaMP6s imaged at 30 Hz. For sufficiently large fields-of-view (FOV), they demonstrated no increase in C1V1-expressing neuron spike rates during imaging; however, subthreshold depolarisation was possible. Mardinly *et al* paired the fast, sensitive actuator Chronos with GCaMP6s [42], and used fast laser scanning to image calcium activity. This reduced the amount of time the imaging light was incident on the opsin expressing cells, and furthermore the fast kinetics of Chronos allowed neurons to repolarise quickly. Although this minimised the spurious depolarisation of Chronos-expressing cells, it did not eliminate subthreshold crosstalk as revealed by voltage-clamp recordings. Finally, as in one-photon, blue actuators have been combined with red-shifted reporters to reduce crosstalk. Forli *et al* paired ChR2 with jRCaMP1a [43], a red-shifted indicator relative to GCaMP6s. Although this combination decreased spurious depolarisation of ChR2-expressing cells, voltage-clamp electrophysiological data showed subthreshold depolarisations. Greater spectral separation between actuators and reporters is needed to eliminate spurious sub- and supra-threshold actuation by readout illumination at wavelengths and intensities that maximise imaging speeds and *S*/*N*.

Here, we have developed a strategy for crosstalk-free readout of neurons expressing the excitatory opsin Chronos [5] with the red light absorbing (peak 650 nm), deep red-emitting (peak 664 nm) calcium reporter CaSiR-1 [44]. Chronos features large photocurrents and millisecond off-kinetics enabling temporally precise action potential stimulation at high repetition rates for both one- [5] and two-photon excitation [15]. Thus, Chronos can drive action potentials in neurons reliably and with millisecond temporal precision, whilst requiring comparatively less light than other opsins.

As Chronos’ blue-green action spectrum is redder than ChR2’s, a readout with orange light-absorbing R-CaMPs, R-GECOs, or spectrally similar synthetic calcium reporters such as Cal-590 [45] would not avoid spurious Chronos actuation, even in one-photon. To overcome the problem of optical crosstalk for readout of neurons expressing Chronos, here we selected a deep red-emitting synthetic calcium indicator, CaSiR-1 (*k*_*d*_  =  0.58 *µ*M [43]), whose absorption spectrum is red and nearly separate from the Chronos action spectrum. We demonstrate the absence of photocurrents in Chronos-transfected Chinese hamster ovary (CHO-K1) cells illuminated with wavelengths and intensities suitable for high *S*/*N* and fast imaging of CaSiR-1 fluorescence transients. Following this, we demonstrate the first all-optical, crosstalk-free activity readout of Chronos-expressing neurons in transgenic mouse brain slices stained with CaSiR-1.

## 2. Methods

### 2.1. Chinese hamster ovary cell culture and transfection

To determine the response of Chronos to imaging wavelengths and intensities suitable for CaSiR-1, we transfected CHO-K1 cells with Chronos and monitored light-evoked transmembrane currents using voltage-clamp electrophysiology. CHO cells were selected to avoid the confounds of the spontaneous activity and numerous active currents present in neurons. Hence, if we were to illuminate the transfected cells and observe any transmembrane currents, these would be caused by opsin activation.

The CHO cells were cultured in Dulbecco’s modified Eagle medium (DMEM)/F-12 medium (21331020, Thermofisher) with the following additions: 10% (volume/volume) fetal bovine serum (F7524, Sigma-Aldrich), 1% (volume/volume) penicillin/streptomycin (P4333, Sigma-Aldrich), and 2 mM L-glutamine (G7513, Sigma-Aldrich).

Before transfection, CHO cells were seeded onto cell-culture treated plastic coverslips (174950, Thermonox). Once the seeded cells reached between 50%–80% confluency, they were transfected with the FCK-Chronos-GFP plasmid (1 *µ*g per 120 000 cells) using a lipofectamine LTX reagent (15338030, Thermofisher). The transfection protocol was optimised to maximise transfection efficiency and final cell health, yielding the following ratio of plasmid to transfection reagents: 1 *µ*g plasmid to 1 *µ*l PLUS reagent to 3 *µ*l lipofectamine LTX reagent. For the entirety of the 4 h transfection period, the culture medium was replaced by opti-MEM reduced serum medium (31985070, Thermofisher), after which the culture medium was changed back to the full DMEM/F-12 media. During the cell culture and transfection periods, the cells were incubated at 37 °C in a 95%/5% O_2_/CO_2_ environment.

We checked the transfection efficiency by imaging the GFP conjugated to Chronos with an inverted immunofluorescence microscope. All electrophysiology and photostimulation experiments were undertaken 24–48 h after transfection to allow enough time for robust Chronos expression.

### 2.2. CHO cell electrophysiology

Once cultured and transfected, we whole-cell voltage-clamped green-fluorescent Chronos-expressing CHO cells using a Multiclamp 700B amplifier (Axon Instruments, Molecular Devices). Membrane currents were digitised with a Power1401 (Cambridge Electronic Devices) controlled by Spike2 software (V5, Cambridge Electronic Devices).

Coverslips with adhered transfected cells were bathed at room temperature in a solution containing (in mM [45]): 140 NaCl, 5 KCl, 10 CaCl_2_, 2 MgCl_2_, 0.3 Na_2_HPO_4_, 0.4 KH_2_PO_4_, 4 NaHCO_3_, 5 glucose, 10 4-(2-hydroxyethyl)piperazine-1-ethanesulfonic acid (HEPES). The osmolality of the solution was adjusted to 300–310 mOsmol kg^−1^ with a 1M sucrose, and the pH was adjusted to 7.3  ±  0.01 with a 1M NaOH. The patch pipette was filled with an artificial intracellular solution containing (in mM [45]): 150 K-gluconate, 2 MgCl_2_, 1.1 ethylene glycol-bis(2-aminoethylether)-N,N,${{{\rm N}}^{\prime }}$,${{{\rm N}}^{\prime }}$-tetraacetic acid (EGTA), 5 HEPES. The osmolality was adjusted to 290 mOsmol kg^−1^, and the pH was adjusted to 7.3  ±  0.01 with 1M KOH.

Patch pipettes were pulled from glass capillaries (PG10150-4, World Precision Instruments) to tips with resistances between 4 and 8 MΩ. Voltage was clamped at  −40 mV. Cells with leak currents of a magnitude greater than 100 pA were excluded from the analysis. We monitored the access and input resistances between photostimulation epochs.

### 2.3. Optical system

**Figure 1.**
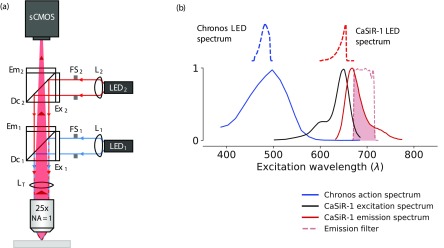
The photostimulation and epifluorescence imaging system (not to scale). (a) A 490 nm LED (LED_1_, blue lines) was collimated by a *f*  =  16 mm aspheric condenser lens (L_1_), and passed through a field stop (FS_1_) before entering a filter cube containing a 475/28 nm excitation filter (Ex_1_), 515 nm long pass emission filter (Em_1_), and 495 nm long-pass dichroic (Dc_1_). A 660 nm LED (LED_2_, red lines) was collimated by a *f*  =  16 mm aspheric condenser lens (L_2_), and passed through a field stop (FS_2_) before entering a filter cube containing a 628/40 nm excitation filter (Ex_2_), 692/40 nm long pass emission filter (Em_2_), and 660 nm long-pass dichroic (Dc_2_). Both LEDs are set up as Köhler illumination paths through a 25×, *NA*  =  1.0 objective. Finally, epifluorescence (solid red area) was collected through the same objective and focussed by a 180 mm tube lens (L_T_) on to an ORCA Flash 4 camera (sCMOS), light direction indicated by dark red arrows). (b) A plot showing the spectral characteristics of the optical system. The blue line on the lower plot shows the action spectrum of Chronos, the black and red lines show the excitation and emission spectra, respectively, of CaSiR-1, and the dashed red line shows the transmission characteristics of the red emission filter (Em_2_, FF01-692/40-25, Semrock). The upper two plots show the spectral distributions of LED light onto the sample, which are the products of the normalised LED emission spectra with the excitation filter percent transmission curves, for Chronos (LED1, dotted blue line) and CaSiR-1 (LED2, dotted red line). The dark red area shows the collected wavelengths of CaSiR-1 fluorescence. (a) [5] (2014) ©. With permission of Springer. (b) Reprinted with permission from [43]. Copyright (2011) American Chemical Society.

Our photostimulation and epifluorescence imaging system integrated red and blue illumination paths. Figure 1 shows the optical path schematic and spectral characteristics. For photostimulation, a 490 nm light emitting diode (LED, M490L4, Thorlabs) was collimated with an *f*  =  16 mm aspheric condenser lens (ACL25416U0-A, Thorlabs) and directed into a filter cube containing a 475/28 nm excitation filter (FITC-EX01-CLIN-25, Semrock), 515 nm long pass emission filter (FF01-515LP, Semrock), and 495 nm long-pass dichroic (FF495-Di03, Semrock). The blue light spectral distribution on the sample is determined by the product of the 490 nm LED’s normalised emission spectrum with the excitation filter transmission curve (figure 1(b)). The peak of this distribution product is 482 nm, and the expected value is 480 nm. This LED was selected as its emission spectrum overlaps with the peak of Chronos’ action spectrum [5] (figure 1(b)). For imaging, a 660 nm LED (M660L4, Thorlabs) was collimated using a second *f*  =  16 mm aspheric lens and directed into a filter cube containing a Cy5 filter set: 628/40 nm excitation filter (FF02-628/40-25, Semrock), 692/40 nm emission filter (FF01-692/40-25, Semrock), and 660 nm long-pass dichroic (FF660-Di02-25x36, Semrock). The red light spectral distribution on the sample is determined by the product of the 660 nm LED’s normalised emission spectrum with the excitation filter transmission curve (figure 1(b)). The peak of this distribution product is 655 nm, and the expected value is 644 nm. This LED and the excitation filter were selected as they result in efficient excitation of CaSiR-1 fluorescence at its peak [43] whilst avoiding the red tail of Chronos’ action spectrum (figure 1(b)).

Imaging and photostimulation were performed through a 25×, *NA*  =  1.0 objective (XLPLN25XSVMP, Olympus). Epifluorescence was imaged by this objective and a 180 mm tube lens (TTL180-A, Thorlabs) onto a scientific complementary metal-oxide-semiconductor (sCMOS) camera (ORCA Flash 4 V2 with Camera Link, 2048  ×  2048 pixels, 6.5 *µ*m pixel size, Hamamatsu). When imaging the green fluorescence from the GFP conjugated to the Chronos, the red filter cube was removed to let green light from the sample onto the camera.

Blue LED power was modulated with an LED driver (LEDD1B, Thorlabs) and triggered with the Power1401 digitiser. For CHO cell photostimulation, the red LED power output was controlled in the same manner. For the brain slice photostimulation and activity readout, we powered the red LED with a precision current controller (SourceMeter 2401, Keithley).

### 2.4. CHO cell photostimulation

Healthy CHO cells were identified using transmitted oblique-contrast infrared light (M780LP1 or M850L3, Thorlabs). Following this, target cells were exposed to a 5 ms blue LED light pulse to check for GFP and consequently Chronos expression. If the cell was both healthy and Chronos-expressing, it was patch clamped as described in section 2.2.

During voltage-clamp recordings we interleaved sets of red (644 nm average wavelength) and blue (480 nm average wavelength) photostimulation trials, beginning with a set of blue pulses to check for the presence of photocurrents. LED intensities stepped between 0.28 and 11.3 mW mm^−2^ were randomised and deployed onto the patched cells, each with a pulse duration of 5 ms. If photocurrents fell to below 90% of the values measured in the first set, this was seen as a marker of declining cell health and/or patch quality, and subsequent trials were excluded from the final analysis. LED control signals and photocurrents were recorded in Spike2, and analysed with custom-written python scripts.

### 2.5. Brain slice preparation and dye loading

Once we determined the response of Chronos to the blue and red LEDs, we next sought to demonstrate CaSiR-1’s efficacy for optically reading out the spiking activity of photostimulated, Chronos-expressing neurons. To this end, we loaded CaSiR-1 acetoxymethyl ester (AM, Goryo Chemicals) onto brain slices expressing Chronos. We photostimulated the neurons with the blue LED whilst imaging with the red LED.

All animal experiments were performed under institutional guidelines, were approved by the United Kingdom (UK) Home Office and were in accordance with the UK Animals (Scientific Procedures) Act of 1986 and associated guidelines. Acute brain slices were prepared from a triple transgenic mouse line (Ai90[TITL-Chronos]-D/Camk2a-tTA/Rasgrf2-2A-dCre [9]) using the protective recovery method [47]. Four-week-old triple transgenic mice were injected with Trimethoprim (TMP), to stabilise the Cre and drive robust expression of Chronos in Layer 2/3 cortical neurons. Mice aged 83 and 357 d were then anaesthetised with isofluorane and decapitated before the brain was immediately removed and placed into ice-cold artificial cerebro spinal fluid (aCSF) containing (in mM): 125 NaCl, 25 NaHCO_3_, 20 glucose, 2.5 KCl, 1.25 H_2_PO_4_, 2 MgCl_2_, and 2 CaCl_2_. The aCSF osmolality was adjusted to 300–310 mOsmol kg^−1^, and the pH adjusted to 7.3–7.4 using 1 M NaOH solution.

We then prepared 400 *µ*m coronal brain slices with a Microtome 7000 (Campden Instruments), whilst the brain was bathed in ice-cold aCSF as described above. Brain slices were then placed into an N-methyl-D-glucamine (NMDG) supplemented aCSF at 36 °C for a 12 min recovery period [47]. The NMDG contained (in mM): 110 NMDG, 2.5 KCl, 1.2 NaH_2_PO_4_, 25 NaHCO_3_, 25 Glucose, 10 MgCl_2_, 0.5 CaCl_2_. The osmolality of this solution was adjusted to 300–310 mOsmol kg^−1^, the pH adjusted to 7.3–7.4 using a 1 M HCl. After the 12 min recovery period, the slices were placed back into aCSF of the same composition of that used during slicing for a 1 h resting period. Both the regular aCSF and NMDG-supplemented aCSF were oxygenated with a 95%/5% O_2_/CO_2_ gas.

Following the 1 h resting period, the slices were loaded with CaSiR-1 AM dye. 50 *µ*g of CaSiR-1 AM was dissolved into a 10 *µ*l dimethyl sulfoxide (DMSO) solution to which we had previously added 10% weight/volume Pluronic F-127 (Invitrogen) and 0.5% volume/volume Kolliphor EL (Sigma-Aldrich). This dye-containing solution was then vortexed to ensure proper mixing. Meanwhile, brain slices were placed in to 2 ml of aCSF before pipetting the dye-DMSO solution onto the surface of the slices, taking care to aim for the cortex as this was the area with Chronos-expressing neurons. The loaded slices were then incubated at 37 °C for 40 min whilst oxygenated by 95%/5% O_2_/CO_2_ gas blowing onto the surface of the solution. This effectively oxygenates the slice without bubble formation in the solution. Finally, the dye-loaded slices were removed and put back into a dye-free aCSF, for a resting period of 20 min before photostimulation and imaging experiments.

### 2.6. Brain slice photostimulation and activity readout

Acute brain slices that both expressed Chronos and were successfully stained with CaSiR-1 were placed under the microscope system described in section 2.3 and submerged in circulating oxygenated aCSF. Layer 2/3 of the cortex was identified and visualised with oblique-contrast infrared imaging. To maximise *S*/*N* for CaSiR-1 imaging, the camera exposure was set to 10 ms, and we increased the red LED intensity to excite fluorescence just below camera saturation in the brightest pixels. To find the subset of cells which were both Chronos-expressing and had taken up CaSiR-1 dye, a ‘pinging’ light pulse consisting of a 3 ms, 1 Hz blue LED pulse with an intensity of 0.063–1.2 mW mm^−2^ was flashed on to the brain slice whilst scanning the cortex laterally and axially throughout the FOV and imaging CaSiR-1 fluorescence. Chronos-expressing, CaSiR-1-loaded cells exhibited fluorescence transients time-synced to the blue LED pings. In some cases, the blue light evoked calcium transients were monitored during perfusion of 1 *µ*M tetrodoxin citrate (TTX, Tocris) in aCSF.

Once Chronos-expressing, CaSiR-loaded cells were identified, we began photostimulation and activity readout trials. Due to the inhomogeneity of the dye loading process, we adjusted the red LED intensity in the range of 0.8–2.26 mW mm^−2^ for each FOV to evoke fluorescence that came close to saturating the camera in the brightest pixels at 100 Hz frame rates, corresponding to an exposure time of 10 ms with a rolling shutter and 512  ×  512 pixels acquired with 4  ×  4 binning. To photostimulate the cells, we reduced the blue LED intensity relative to the pinging signal to find the minimal intensity which still produced calcium responses, which ranged from 0.054 to 1.2 mW mm^−2^. We needed to use much higher blue light intensities in slices prepared from younger mice, possibly because the density of Chronos molecules in neurons increased with age. The FOV was then stimulated with three, 3 ms flashes of the blue light, delivered at frequencies of 0.5 Hz, 1 Hz, 2 Hz, 5 Hz, 10 Hz, 15 Hz, and 20 Hz. The red imaging LED was on throughout each trial, and off between trials to avoid excess CaSiR-1 photobleaching. We began the trials at the lower stimulation frequencies and continued to higher frequencies if the cell(s) remained responsive. Finally, for each imaging trial, we interleaved a trial in which the red LED was off whilst deploying blue light onto the brain slice. This was to monitor for imaging artefacts caused by bleed through of the blue light on to the camera, or activation of the CaSiR-1 with the blue photostimulation light. We used these ‘blue-only’ trials to quantify and subtract such artefacts from our imaging data. We calculated *dF*/*F* using the following formula:

\begin{align*} \newcommand{\e}{{\rm e}} \displaystyle \frac{\Delta F}{F}=\frac{F-{{F}_{0}}}{{{F}_{0}}-{{F}_{dark}}},\nonumber \end{align*}

where $F$ was the raw fluorescent signal (in counts), ${{F}_{0}}$ was the baseline fluorescence taken as the average fluorescence in the epoch preceding photostimulation (counts), and ${{F}_{dark}}$ was the signal from the camera in the dark (counts).

The signals were extracted from the imaging trials by manually selecting a ROI over the cell of interest using Image-J. Further analysis was performed in custom written Python code. The signals contained flat averages and remained consistent across analysis of the same cell. To quantify what portion of the ROI peak signal was contributed from surrounding cells, or ‘background’, we subtracted the average signal from a donut shaped ROI surrounding each cellular ROI. Trials in which and after which the peak cellular ROI signal was equal to background, indicating that the cell was no longer responding, had died or was bleached, were excluded from cellular response statistics.

## 3. Results

### 3.1. Wavelengths suitable for CaSiR-1 imaging do not actuate Chronos-mediated photocurrents

**Figure 2.**
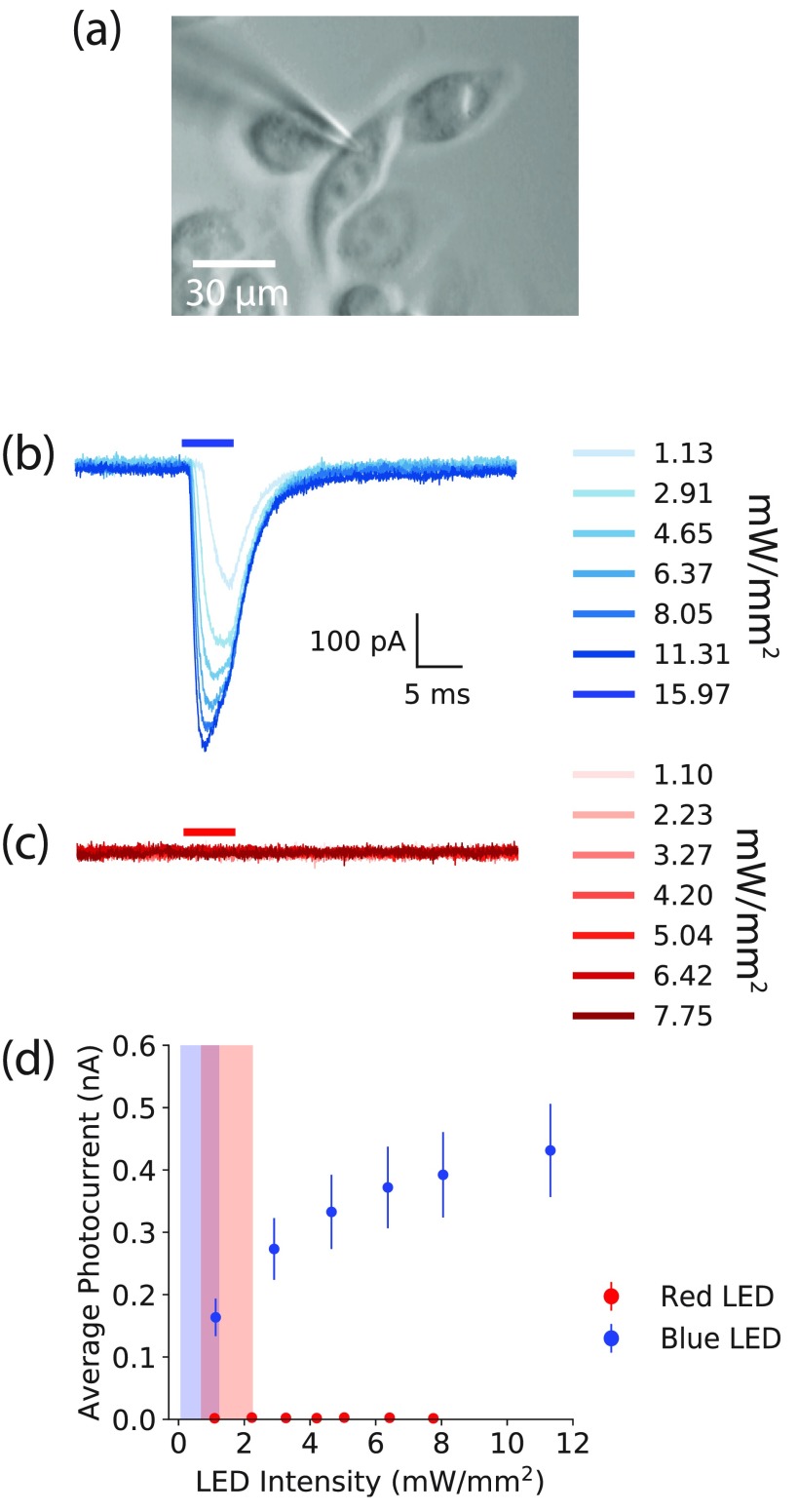
Blue light, but not red light pulses, evoke photocurrents in Chronos-expressing CHO cells. Voltage clamp, whole cell recordings of a Chronos-expressing CHO cell (a) when stimulated with (b) blue light and (c) red light, both at increasing illumination power densities. The horizontal blue and red lines above the voltage-clamp traces represent 5 ms long red and blue LED pulses incident on the patched cell respectively. (d) The average induced photocurrent of Chronos-expressing cells increased with the blue LED power density, but remained zero with increased red LED power density. The graph shows the average currents measured in *N*  =  4 cells, across 16 trials for blue light, and 13 trials for red light. Vertical lines represent the standard error of the mean (S.E.M); S.E.M values for the red light data series are too small to be seen on this scale. The blue shaded area represents the power densities used to photostimulate Chronos-expressing brain slices; the red shaded area represents the range of power densities used to image CaSiR-1 fluorescence in the brain slice preparations.

We measured transmembrane currents in Chronos-transfected CHO cells with whole cell voltage clamp, in response to 5 ms red and blue light pulses (figure 2(a)). Blue light pulses ranging from 1.1 to 11.3 mW mm^−2^ evoked photocurrents which increased with increasing intensity (figures 2(b) and (d)). The rate of increase in photocurrents decreased with increasing intensity with opsin saturation. Red light pulses, however, evoked no measurable photocurrents (figures 2(c) and (d)), across all measured intensities from 1.1 to 7.8 mW mm^−2^. The intensity ranges tested for both blue and red sources exceeded the intensities needed to stimulate and image neuronal activity (indicated on figure 2(d)) in the following section by several fold. As crosstalk would only increase with intensity, this indicates that imaging with the red light in the subsequent all-optical stimulation and readout of Chronos-expressing brain slices would evoke no spurious photocurrents.

### 3.2. Crosstalk-free readout of Chronos-expressing neurons with CaSiR-1

**Figure 3.**
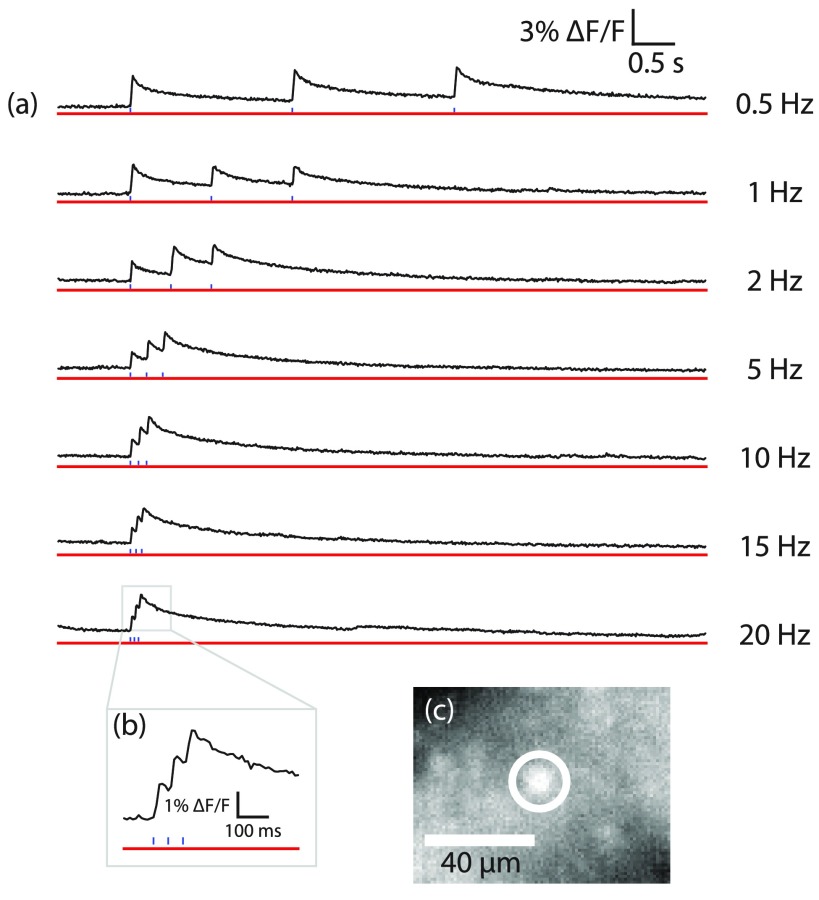
By staining Chronos-expressing brain slices with the deep-red calcium reporter CaSiR-1, we were able to optically image neuronal activity stimulated up to 20 Hz without crosstalk at 100 frames s^−1^. (a) A set of CaSiR-1 fluorescence imaging traces recorded from a single cell (c) in single trials. The horizontal red lines represent imaging epochs during which the red LED illumination power density was 1.85 mW mm^−2^. Vertical blue lines indicate the timing of 3 ms blue LED flashes incident on the CaSiR-1 loaded, Chronos-expressing neurons. Each blue light flash had a power density of 0.063 mW mm^−2^. (b) A zoomed-in window showing the CaSiR-1 imaging trial whilst photostimulating the brain slice with blue light at 20 Hz. We observed distinct calcium responses for each blue LED light flash. Imaging data were acquired at 100 Hz frame rates. Video of the calcium response is included in the supplementary information. (c) An image showing the cell and ROI (white circle) over which the traces in (a) were averaged. The frame was taken at the peak of fluorescence response of the cell following a blue light flash.

After determining that illuminating Chronos-expressing CHO cells with red light suitable for CaSiR-1 imaging did not evoke any photocurrents, we demonstrated crosstalk-free functional imaging of Chronos-expressing neurons in acute brain slices. At 100 Hz frame rates, we resolved in single trials calcium transients evoked by 3 ms blue light pulses (0.054–1.24 mW mm^−2^, figure 3, see also supplementary video 1 (stacks.iop.org/JPhysD/52/104002/mmedia)) from five cells in two mice. Calcium transients from these cells could be imaged for between 3 and 90 blue stimulations. We repeated the blue light pulse photostimulation at frequencies ranging from 0.5 to 20 Hz. Even at the highest frequencies, we were able to resolve calcium responses from each individual LED pulse of the trial (figure 3(b)). We were able to record transients in response to stimulation for up to 50 min in one cell. In 28 trials of 3 blue light stimulations each across five cells in two mice, the peak signal after spatial averaging over 113–227 pixels had a median of 1.56% *dF*/*F*, with 90th and 10th percentiles of 1.17% and 2.88%. The median noise level was 0.07%, with 90th and 10th percentiles of 0.09% and 0.06%. The median baseline photon flux was 11 461 200 photons pixel^−1^ s^−1^, with 90th and 10th percentiles of 11 772 706 and 11 163 807 photons pixel^−1^ s^−1^ (pixel size 1.04 *µ*m). The median *S*/*N* was 22.4, with 90th and 10th percentiles of 43.0 and 14.6. The cellular ROIs bleached an average of 0.036% s^−1^. *dF*/*F*, with 90th and 10th percentiles of  −0.011% and  −0.069% s^−1^. The peak signal from a donut shaped ROI, or ‘background’, surrounding the cell after spatially averaging over 1075–1810 pixels had a median of 0.92% *dF*/*F*, with 90th and 10th percentiles of 1.49% and 0.68%. The background signal was subtracted from that of the cell ROI to give the background subtracted peak response with median of 0.71% *dF*/*F*, with 90th and 10th percentiles of 1.46% and 0.14%. In this widefield imaging configuration, over half of the median cellular ROI peak signal was contributed by surrounding cells.

The slow decay of evoked calcium transients results from a combination of the time for calcium to return to baseline concentrations following an action potential and CaSiR-1 dissociation kinetics. It is important to note that the electrical action potentials causing these calcium rises are brief, lasting ~1 ms. In addition, each blue light pulse may have evoked one or more action potentials, each contributing to the measured calcium transient peak. Bath application of 1 *µ*M TTX reduced the slowly decaying blue light evoked calcium transients after 15 min to brief, small transients, <5% of the peak pre-TTX transient amplitude and lasting only as long as the 3 ms blue light pulses (figure S1). These small residual transients, which were not caused by action potentials, could potentially reflect calcium conductance directly through Chronos channels and/or low voltage activated calcium channels.

To quantify crosstalk or bleedthrough caused by the blue photostimulation light, we acquired trials in which blue light was pulsed onto the sample in the absence of red light, called ‘blue only’ trials. This crosstalk does not affect cell physiology and appears as increased image counts during blue light on periods. As the stimulation period is known, the artefact can be simply subtracted. Averaged over the entire FOV, these ‘blue only’ trials revealed artefacts with a median pixel count of 1.7 digital number (DN), with 90th and 10th percentiles of 115 and 0.94 across 105 trials. The cells in the younger mouse required approximately 10 times higher stimulation intensities compared to the older mice, resulting in larger stimulus artefacts. The artefacts in the old mouse corresponded to 3.8% of the average baseline noise (standard deviation) of trials during which the red light was on, and 0.2% of the average peak calcium transient amplitude. In the young mice, the artefacts were around 4 times larger than the RMS noise level of the fluorescent traces. Due to the sCMOS rolling shutter, these artefacts may be underestimated by, at most, a factor of two due to the blue light pulse spanning two adjacent frames. Even if multiplied by two, these artefacts were small relative to the measured calcium transients.

We recorded non-Chronos evoked calcium transients from many cells at a variety of depths. We measured the depths of a subset of active cells (*n*  =  14), and the median was 47.5 *µ*m, with 90th and 10th percentiles of 66.5 and 33.5 *µ*m. We did not record from FOV with multiple blue light-responding cells, however we did record from areas with up to 30 active cells.

## 4. Discussion

We have demonstrated the first crosstalk-free functional readout of Chronos-expressing light-actuated neurons. We first established that the red wavelengths used to excite CaSiR-1, at intensities greater than three-fold higher than those needed to image its fluorescence in stained brain slices at 100 frames s^−1^, evoked no photocurrents in voltage-clamped Chronos-expressing CHO cells. This indicates that the red light used to excite CaSiR-1 in brain slices most likely did not evoke spurious Chronos-mediated depolarisations, sub- or supra-threshold. This crosstalk-free readout is made possible by the spectral separation of the Chronos action spectrum from the CaSiR-1 absorption spectrum, which is larger than previously reported pairings of opsins and calcium reporters. Although not shown here, CaSiR-1 could also be used for crosstalk-free one-photon imaging of ChR2 or CheRiff transfected neurons, whose action spectra are bluer than Chronos’.

Identifying crosstalk-free combinations of opsin actuators with activity reporters will be crucial for future studies of neural networks free from the confounds of unintended sub- or supra-threshold depolarisation. The benefits of activity reporters which can be efficiently excited by red light are fourfold. First, as shown here, red light excitation can be used to avoid spurious activation of blue to green light-sensitive opsins. Second, longer reporter excitation and emission wavelengths are less scattered than blue or green light, enabling deeper imaging in mammalian brains. Third, intrinsic cellular fluorophores absorb red and deep red wavelengths less than bluer wavelengths, resulting in less background autofluorescence [48]. Finally, red wavelengths occupy a window where both water and haemoglobin absorbance are low relative to other wavelengths, decreasing tissue heating and damage [48].

At present, no genetically encoded calcium indicators are available with absorption spectra red-shifted enough to avoid overlap with blue to green light-sensitive opsins. Furthermore, CaSiR-1 is currently the only commercially available red absorbing calcium reporter conjugated to an AM-ester for easy neuronal bulk-loading. However, other synthetic dyes or genetically encoded activity reporters may become available [48]. For example, Cal-670 (AAT Bioquest) is a calcium reporter which has a red absorbance and deep red emission spectrum, but is not currently available as an AM-conjugated dye. Alternatively, synthetic and genetic voltage reporters, such as QuasArs and Archons, are being developed which are efficiently excited by red wavelengths [24, 30]. These too may be suitable for crosstalk-free activity read out of blue to green light-sensitive opsins like Chronos, ChR2, and CheRiff. However, it is important to note that the intensities used to excite the red-absorbing QuasAr and Archon membrane potential reporters are 100–1000 fold the intensities we tested here in the Chronos-expressing CHO cells. Red light illumination at these higher intensities may indeed actuate spurious currents in blue light sensitive opsins, and future work could test this directly.

Although we were able to demonstrate crosstalk-free activity readout of Chronos-expressing cells, we observed imaging artefacts potentially caused by either bleed through of the blue LED light on to the camera sensor, or activation of the CaSiR-1 with the blue LED light. The artefacts ranged from less than 1 count to 115 counts, depending on the intensity of blue light required to stimulate the cell. In any case, this artefact was confined to a single frame, could be corrected through simple subtraction, and did not affect the physiology.

Future investigation into neuron health and electrophysiological properties as a function of opsin expression, dye loading, illumination intensity, duration and repetition would be of high practical value for developing experimental protocols combining CaSIR-1 and Chronos. Cell health in slices varies widely as a function of slice quality. In addition, it was recently noted by Daigle *et al* [46] that driving the expression of Chronos under the TIGRE1.0 transgene system with a lower expressing ROSA26-ZtTA instead of the higher expressing Camk2a-tTA results in cells that appear healthier. As such there is an important trade-off to be quantified between expression level and cell health.

The work conducted here used single photon excitation both for the Chronos actuator and CaSiR-1 calcium reporter. Future work is needed to characterise the two-photon excitation spectrum of CaSiR-1, and determine where it overlaps with the two-photon action spectrum of Chronos. At present, ultrafast lasers do not routinely reach wavelengths suitable for such characterisations, although this will change as suitable lasers and/or optical parametric amplifiers and oscillators in the 1200–1500 nm range become cheaper and more accessible. Identification of CaSIR-1 or other spectrally similar calcium reporters with large two-photon cross-sections would enable optical sectioning, scattering robust imaging of Chronos-expressing neurons with little or no crosstalk.

Widefield blue light excitation of the Chronos opsin, while simple and inexpensive, precluded validation of whether the CaSIR-1 transients resulted from direct actuation of Chronos in these same cells, or through direct and/or synaptic stimulation of all expressing cells in the slice. Future studies can address this through whole-cell patch clamp or by patterning light onto selected cells with one- or two-photon computer generated holography or generalised phase contrast modalities (reviewed by [47]), which have recently demonstrated the exciting capability to target specific neurons distributed in 3D [48–50]. The combination of Chronos or other blue opsins with fluorescent reporters like CaSIR-1 efficiently excited above 640 nm could thus enable all-optical interrogation of 3D neuronal networks free of crosstalk both below and above action potential threshold.
